# Phase transition mechanism and bandgap engineering of Sb_2_S_3_ at gigapascal pressures

**DOI:** 10.1038/s42004-021-00565-4

**Published:** 2021-09-02

**Authors:** Zhongxun Cui, Kejun Bu, Yukai Zhuang, Mary-Ellen Donnelly, Dongzhou Zhang, Philip Dalladay-Simpson, Ross T. Howie, Jiandong Zhang, Xujie Lü, Qingyang Hu

**Affiliations:** 1 Center for High Pressure Science and Technology Advanced Research, Shanghai, P.R. China; 2 Key Laboratory of Metallogenic Prediction of Nonferrous Metals and Geological Environment Monitor, Ministry of Education, Central South University, Changsha, P.R. China; 3 Hawai’i Institute of Geophysics and Planetology, School of Ocean and Earth Science and Technology, University of Hawai’i at Manoa, Honolulu, HI USA; 4 CAS Center for Excellence in Deep Earth Science, Guangzhou Institute of Geochemistry, Chinese Academy of Sciences, Guangzhou, P.R. China

**Keywords:** Density functional theory, Chemical bonding, Chemical physics, Solid-state chemistry

## Abstract

Earth-abundant antimony trisulfide (Sb_2_S_3_), or simply antimonite, is a promising material for capturing natural energies like solar power and heat flux. The layered structure, held up by weak van-der Waals forces, induces anisotropic behaviors in carrier transportation and thermal expansion. Here, we used stress as mechanical stimuli to destabilize the layered structure and observed the structural phase transition to a three-dimensional (3D) structure. We combined in situ x-ray diffraction (XRD), Raman spectroscopy, ultraviolet-visible spectroscopy, and first-principles calculations to study the evolution of structure and bandgap width up to 20.1 GPa. The optical band gap energy of Sb_2_S_3_ followed a two-step hierarchical sequence at approximately 4 and 11 GPa. We also revealed that the first step of change is mainly caused by the redistribution of band states near the conduction band maximum. The second transition is controlled by an isostructural phase transition, with collapsed layers and the formation of a higher coordinated bulky structure. The band gap reduced from 1.73 eV at ambient to 0.68 eV at 15 GPa, making it a promising thermoelectric material under high pressure.

## Abstract (web summary)

Antimonite (Sb_2_S_3_) has potential applications for solar energy, but how its layered structure changes under pressure is incompletely understood. Here diamond anvil cell experiments supported by first principles calculations offer a structural explanation for experimentally observed phase transitions.

## Introduction

The needs for environmentally friendly and sustainable energy supplies are the prerequisites for achieving carbon neutrality. According to Renewables 2020 Global Status Report, renewable technologies like solar, geothermal, and wind power have provided 8.7% of the final world’s primary energy consumption^1^. The development of solar cells has enjoyed its blossom since the last decade^2–5^. However, their broad implications are still restricted by the materials cost, reliability, and power conversion efficiency (PCE)^6^. Sb_2_S_3_ is a promising solar energy absorber with affordable cost, good Earth abundance and non-toxic composition^7–9^. It features a relatively low melting point (550 °C), which helps to synthesize high quality film at below 350 °C^10^. In particular, Sb_2_S_3_ has a high absorption coefficient (*a* > 10^4^ cm^−1^)^11^, and the band gap of 1.7–1.8 eV matching the required *E*_g_ values of Si-based solar cells, making its maximum theoretical PCE above 40%^12,13^. However, its performance is dragged by self-trapping states, which limits the upper approximately maximum open circuit voltage at around 0.8 V and thus, its realistic PCE is still lower than 16%^9,14–17^.

Sb_2_S_3_ is also regarded as a promising thermoelectric material^18^. Sb_2_S_3_ features a large Seebeck coefficient and relatively low lattice thermal conductivity due to varied activity of Sb electron lone pairs and soft Sb–S bonds^18,19^. However, on the basis of the intrinsic correlation between the Seebeck coefficient *S* and the electrical conductivity *σ*, the large band gap of pure Sb_2_S_3_ (1.73 eV) makes it difficult to achieve a significant increase in the power factor^20^. Energy band engineering, for instance, doping^21–23^ and applying strain^24^, have been used to decouple *S* and *σ* in order to achieve higher power factor and conversion efficiency. Specifically, applying pressure stiffens bonds and alters electronic structures, which can be used as an environmental-friendly method to engineer band gap energy.

Pressure by applying stress directly alters bonding distances, engineers bandgap energies and has recently been used to improve photon efficiencies in many materials^5,17,25–27^. Sb_2_S_3_ is an archetypal layered structure that contains parallel Sb_4_S_6_ chains formulated in 2 × 1 crumpled sheets^28^. Two major pressure induce phase transitions were reported below 15 GPa^29^, despite existing arguments. Within 4–5 GPa, Sorb et al.^30^ reported that Sb_2_S_3_ underwent an isostructural electronic topological phase transition. The results were later confirmed by Dai et al.^31^ and Efthimiopoulos et al.^32^ who also found the transition incurred the redistribution of Sb^3 + ^lone-electron pairs. However, Ibañez et al.^33^ reported that no evidence from the first-principles simulation would support electronic topological transition below 10 GPa. At higher pressures of 10–15 GPa, more controversies regarding the second phase transition processes were observed. Efthimiopoulos et al.^32^ suggested pressure-induced structural disordering took place at 15 GPa and completed at ~20 GPa, while theoretical studies show Sb_2_S_3_ is stabilized in a quasi-3D structural phase transition at similar pressures^33^. Upon futher compression, Sb_2_S_3_ becomes a substituinal alloy^34^. Since the crystal structure strongly influences the electronic band gap of materials, it is necessary to conduct an updated study and figure out the underlying transition mechanisms.

In this work, we focus on the evolution of the layering structure in Sb_2_S_3_ in response to external stress. Detailed structural analysis of Sb_2_S_3_ was performed up to 20 GPa, where we found the (Sb_4_S_6_)_n_ were completely collapsed. At the same time, we measured the evolution of band gap energies using ultraviolet-visible absorption spectroscopy. Based on experimental results and first-principles simulation using hybrid functionals, we revisited the sterically controlled phase transition at ~4 GPa and 11 GPa. We observed two major discontinuities in band-gap energies, corresponding to the two above mentioned transition points. The transition mechanism can be defined by the onset of a direct-band gap type at ~4 GPa and a fully developed 3-dimentional (3D) Sb_2_S_3_ crystal structure at above 11 GPa.

## Results and discussion

### Crystal structure of compressed Sb_2_S_3_

**Fig. 1:**
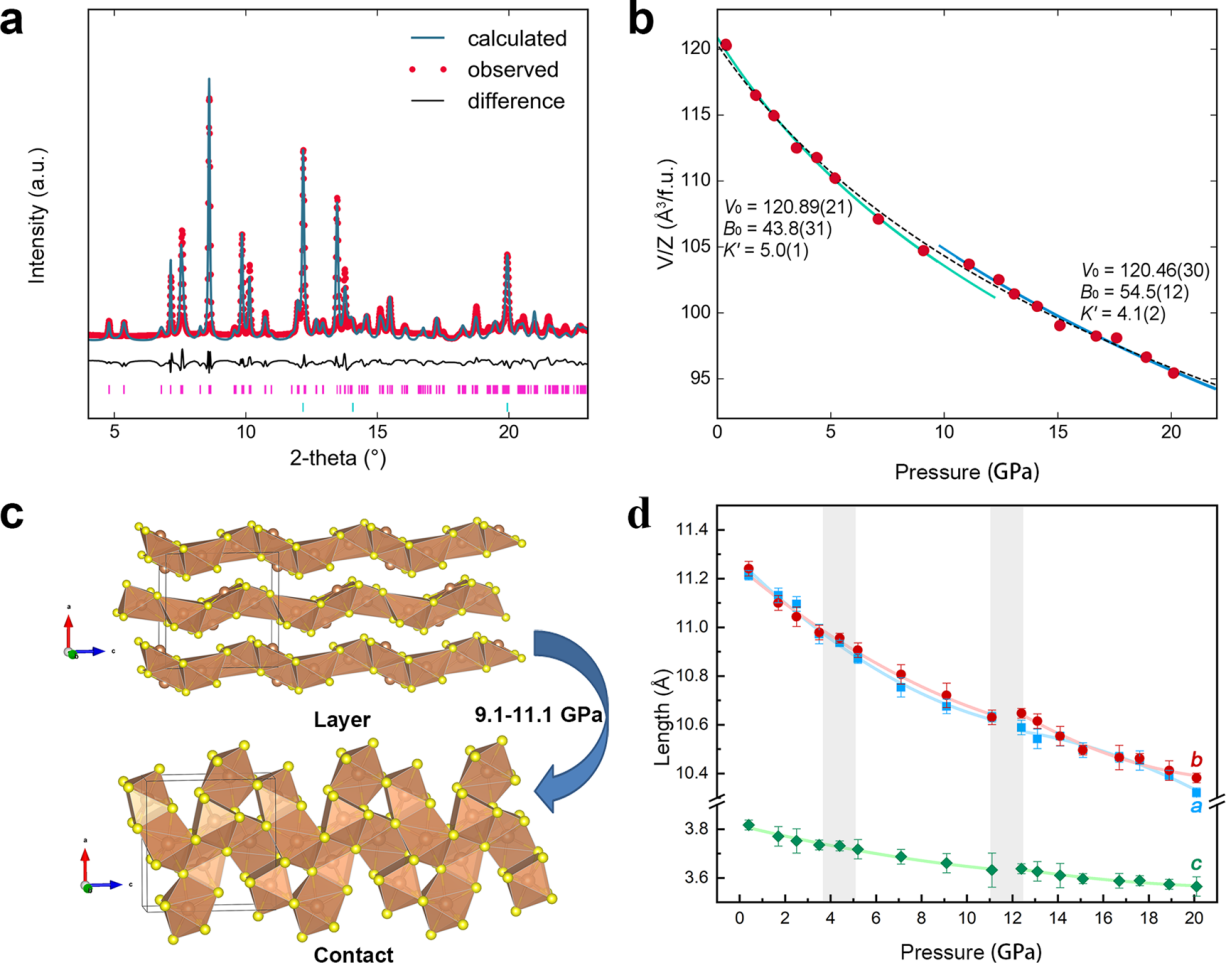
XRD and EOS fitting result. **a** Rietveld refinements results of Sb_2_S_3_ at 11 GPa. **b** *P*–*V* data is fitted to the third-order Birch-Murnaghan equation. Solid lines were fit to two segments of data while dashed line was fit for the whole range of data with *V*_*0*_ = 120.3(7) Å^3^/f.u.*, K*_0_ = 48(6) GPa and* K*′ = 5.4(9). Volume uncertainties are generally smaller than the size of symbols. **c** the signature layered chains of SbS_5_ polyhedral collapsed into a bulky structure in between 9.1 and 11.1 GPa. **d** Evaluation of crystal structure parameters up to 20.6 GPa. The errorbars in cell volume and lattice parameters were generated by the refinement of X-ray diffraction pattern and the Unitcell fitting process.

Previous studies on the layered structure of Sb_2_S_3_ suggested a set of pressure-induced polymorphic and electronic phase transitions. Here, our first experiment mainly focused on the structural properties of single-crystal Sb_2_S_3_. At pressures below 10 GPa, the sample is readily resolved in the conventional way of single-crystal crystallography (e.g., at 1.7 GPa, Table 1). The rocking curves of representative single-crystal diffraction spots became flattened at higher pressure. For data collected above 10 GPa, single-crystal refinement is no longer feasible due to twinning and crushed samples, but we can still treat the patterns as pseudo-Debye-Scherrer rings, which can be resolved using the Rietveld method. We noticed diffraction peaks in the integrated diffraction patterns are consistent through the studied pressure range (Supplementary Fig. 1), indicating Sb_2_S_3_ kept the same *Pnma* space group up to 20.1 GPa. However, by comparing the atomic positions, S atoms involve greater atomic displacement and form new bonds with neighboring Sb atoms (Table 1 and Fig. 1). Consequently, the signature layered chains of SbS_5_ polyhedral collapsed into a 3D crystal structure within 9.1 to 11.1 GPa, where an isostructural phase transition might have taken place (Fig. 1c).

**Table 1:** Resolved atomic positions for Sb_2_S_3_ at 1.7 and 11.1 GPa.

Run	Sb_2_S_3_ (1.7 GPa)	Sb_2_S_3_ (11.1 GPa)
Temperature	297 K	297 K
Space group	*Pnma* (#62)	*Pnma* (#62)
*a* (Å)	11.276 (4)	10.531 (2)
*b* (Å)	11.002 (2)	10.501 (5)
*c* (Å)	3.744 (1)	3.7511 (3)
*V* (Å^3^)	464.48 (22)	414.79 (21)
*Z*	4	4
*V/Z* (Å^3^ / f. u.)	116.12 (4)	103.7 (4)
Atom position	*x, y, z*	*x, y, z*
Sb_1_	0.3258 (13), 0.4728 (5), 0.25	0.3135 (6), 0.5234 (12), 0.25
Sb_2_	0.0363 (2), 0.3491 (5), 0.75	0.0430 (7), 0.3268 (14), 0.75
S_1_	0.1928 (5), 0.2939 (14), 0.25	0.2279 (18), 0.2441 (29), 0.25
S_2_	0.1240 (7), 0.5556 (15), 0.75	0.1571 (20), 0.546 (4), 0.75
S_3_	0.4391 (5), 0.3718 (13), 0.75	0.4012 (22), 0.394 (4), 0.75

At 1.7 GPa, Sb_2_S_3_ is the original layered structure. The sample undergoes an isostructure phase transition, and the group movement of S atoms mainly contribute to the build-up of the 3D structure at high pressure. Details of single-crystal refinement are in Supplementary Table 2.

It is possible to fit the whole range of data into one equation of state (dashed line in Fig. 1b). However, the collapse of interlayer space motivated us to fit the *P*–*V* data to two segments, namely before and after the isostructural phase transition. Therefore, we used two sets of EOS parameters. To characterize the isostructural phase transition, we used the third-order Birch-Murnaghan equation^35^ to fit the cell volumes over pressure and studied the equation of state:

1 $$P\left( V \right) = \frac{{3B_0}}{2}\left[ {\left( {\frac{{V_0}}{V}} \right)^{\frac{7}{3}} - \left( {\frac{{V_0}}{V}} \right)^{\frac{5}{3}}} \right]\left\{ {1 + \frac{3}{4}\left( {B^\prime - 4} \right)\left[ {\left( {\frac{{V_0}}{V}} \right)^{\frac{2}{3}} - 1} \right]} \right\}$$

where *B*_0_ and *V*_0_ are the bulk modulus and volume at ambient pressure, *V* is the deformed volume under pressure, and *B*′ is the derivate of the bulk modulus with respect to pressure. In addition, we plot the Eulerian strain-normalized pressure (*F*–*f*) relation in Supplementary Fig. 2 and the evolution of interlayer distance in Supplementary Fig. 3. A clear stiffening of lattice was observed near the isostructural transition point, where the *B*_0_ of reconstructed 3D-Sb_2_S_3_ is 24% greater (*B*_0_ = 54.4 GPa compared to 43.8 GPa for the low-pressure phase). Seeing from the evolution of structural parameters (Fig. 1c, d), the compression in low-pressure phase is mainly achieved by squeezing layers made of Sb–S polyhedral chains. Above the transition point, the material becomes less-compressible as the Sb_2_S_3_ features higher atomic coordination described in the 3D structure. A notable jump of the ***c*** axis length is observed at 11 GPa, accompanying the shortening of ***a*** and ***b*** lattice axis and the isostructural transition. This compression anomaly is the signature for the formation of new chemical bonds, which may profoundly change the electronic structure of Sb_2_S_3_. Pressure-regulated dynamic stereochemistry has been reported in many layered materials^36–41^. We also characterized the interlayer distance as a function of pressure force (Supplementary Fig. 3 and Supplementary Data 1), which suggest the layer is initially maintained by the van der Waals force and then by covalent bonding upon isostructural transition.

**Fig. 2:**
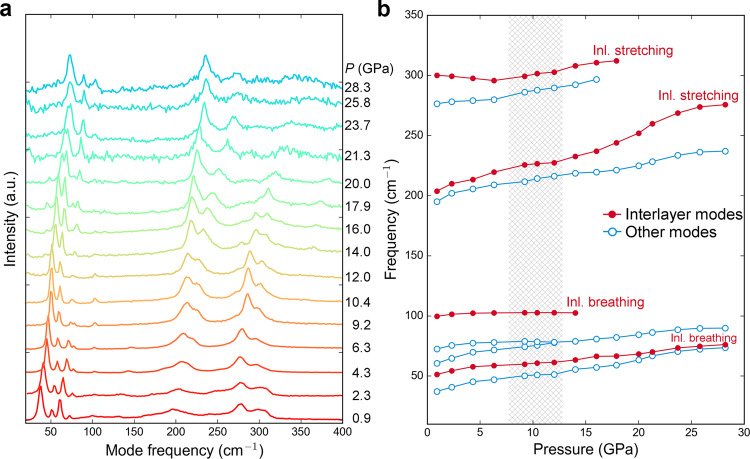
Low-frequency Raman spectroscopy to 28.3 GPa. **a** Raman spectra of compressed Sb_2_S_3_ in the range of 0–400 cm^−1^ wavelength. The asterisk peaks were not traceable thus not assigned to specific modes. **b** Evolution of each Raman mode over pressure. The frequency of peak has maximum uncertainty of 5 cm^−1^. Mode displacements are derived from the first-principles simulation at low pressure. Inl interlayer.

Our x-ray diffraction analysis indicate that the isostructural phase transition is achieved by the reconstruction of bonds and coordination environments. In light of this, we performed Raman spectroscopy which is more sensitive to the change of chemical bonds. The experimental results are summarized in Fig. 2 and compared with the first-principles simulation (Supplementary Fig. 4). The frequencies of modes in our experiments generally agree with the previous experiment by Dai et al.^31^ and Efthimiopoulos et al.^32^ Here, we identified each mode on the basis of first-principles calculations and classified them into two groups: interlayer modes (solid red circles in Fig. 2b) and other bulk-like inner-layer modes (open blue circles in Fig. 2b). The interlayer breathing mode ~100 cm^−1^ was terminated soon after the transition, suggesting the completion of the layer to bulk isostructural phase transition. While both interlayer stretching modes were observed to be softened below ~14 GPa, they robustly shifted to higher frequencies upon the completion of the phase transition. The rest bulk-like inner-layer mode exhibit blueshift over the pressure region. It was also reported that a turning point of electronic conductivity was seen at ~5 GPa^31^. However, our XRD and Raman experiments alone were unable to identify this phase transition. The electronic phase transition is unlikely due to the collapse of Sb_2_S_3_ layers, but may due to changes in the electronic band structures.

### Ultraviolet–Visible and bandgap analysis

**Fig. 3:**
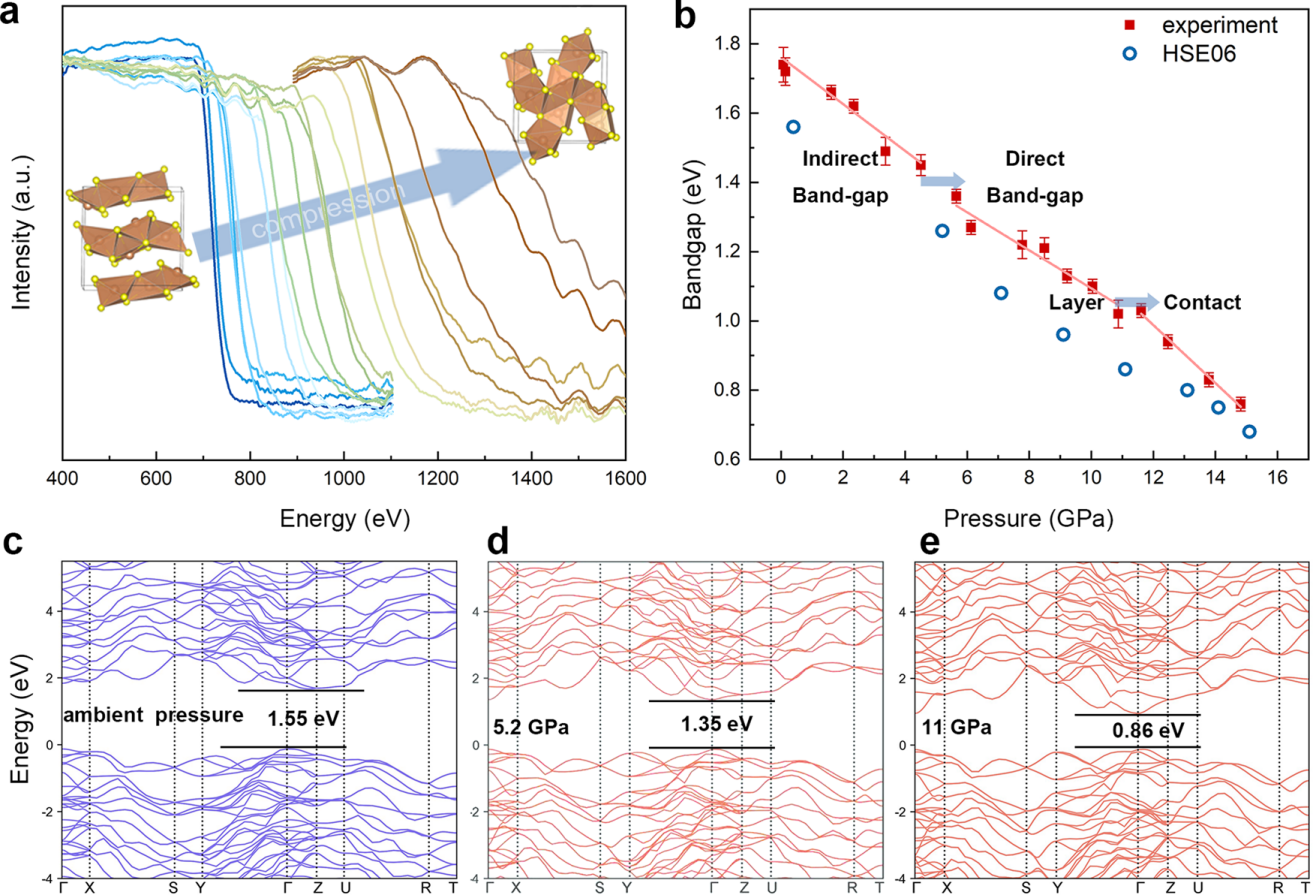
UV-vis absorbance data to 14.8 GPa. **a** Results of absorbance from room pressure to 14.8 GPa. **b** Bandgap fitting by K-M equation (red) and calculated by DFT with HSE06 (blue). **c** Electronic structure of Sb_2_S_3_ with bandgap 1.55 eV at ambient pressure. **d** At 5.2 GPa, Sb_2_S_3_ is converted into a direct bandgap semiconductor with bandgap 1.35 eV. **e** Sb_2_S_3_ undergoes a 2D-3D phase transition, and the bandgap is compressed to 0.86 eV at 11 GPa.

Therefore, we are motivated to measure the electronic band-gap energy in compressed Sb_2_S_3_ single crystals. At ambient condition, bulk Sb_2_S_3_ has indirect band-gap energy of 1.73 eV, which is suitable for solar cell application, although its self-trapping states substantially inhibit its photon-energy conversion^6,14,42^. Here, we obtained the ultraviolet-–visible (UV–Vis) spectra and calculated the bandgap by the Kubelka–Munk (K–M) equation^43^ over the regime of phase transition (Fig. 3a, b and Supplementary Data 1). We also conducted the first-principles calculation on the basis of HSE06 hybrid functionals to find the mechanism. The calculated band-structure confirmed that ambient Sb_2_S_3_ has an indirect semiconductor, with its valence band maximum located at Γ (0, 0, 0) and conduction band minimum (CBM) located near Z, at (0,0,0.375) (Fig. 3c). However, the CBM moved to the Γ (0, 0, 0) point of the Brillouin zone once pressure was increased to 5.2 GP and Sb_2_S_3_ tuned into a direct bandgap semiconductor. Guided by our simulation results, the bandgap change induced the first discontinuity in our bandgap measurement. Such electronic phase transition was also predicted in layered structures like MoS_2_^44^ and was known to boost electrical conductivity^45^. Similarly, the onset of direct band-gap type would be the principal reason of observed higher electrical conductivity in Sb_2_S_3_.

Once we pressurized the sample to the previously observed isostructural transition point, we observed a kink of band-gap energies (Fig. 3b). It is worth noting that the isostructural phase transition does not alter the direct bandgap, but only stagger the change of bandgap energies. The kink was verified by our first-principles simulation, where the closing of band gap slightly accelerated in between 11 and 13 GPa. It is worth noting that the narrowed bandgap at above 11 GPa is specifically useful for thermoelectrical materials^20^. We stopped our experiment at ~15 GPa because the band gap energy becomes greatly lowered and evolves towards full metallization^31^. A previous work on Sb_2_S_3_ thin film reported a broad albeit weak photoluminescence peak from the states at ambient conditions^14^. We also closely monitored possible photoluminescence during the compression experiment through our optical window. However, throughout the pressure experiment, the photoluminescence has yet become visible and should keep weak due to the active self-trapping states.

### Collapsed layers and the bandgap transition

On the basis of pioneering works, our results attempt to clarify two main points during the densification of Sb_2_S_3_ crystals. First, the previously observed turning of electrical conductivity at ~4 GPa^31^ is due to the shift of CBM to the Γ point of Brillouin zone. At the same pressure, Sb_2_S_3_ is still chained by Sb–S polyhedron and a well-defined layered structure. Also, our experiments demonstrate that compressed Sb_2_S_3_ maintains the same crystal space group up to 20.1 GPa under room-temperature compression. Although under high pressure, the *Pnma* phase might not be the most energetically favored phase^34^, large-scale atomic movement or reconstructive structural transition was not observed in our single-crystal experiment most likely due to the high energy barrier, the bulky single-crystal sample and relatively fast compression rate. Transition kinetics and sample crystallinity should be taken into consideration for polymorphism in similar layered structures^46,47^.

Loading strain is well-known to influence the electrical conductivity of materials, and may reversely response to the Seebeck coefficient. Our experiment demonstrated that hydrostatic compression of Sb_2_S_3_ single-crystals has greatly squeezed the interatomic spacing and reduced the bandgap to below 1.0 eV above 11 GPa, at which pressure normal strains of 5.89%, 6.11%, and 5.42% were applied along the *x*, *y*, and *z* axis, respectively. The improved electrical conductivity may partially offset the relatively low carrier concentration of Sb_2_S_3_, which was measured on the order of 10^12^ at ambient conditions and enhanced the overall thermoelectric performance. However, it is still necessary to directly calibrate the Seebeck effect under pressure for more accurate description under pressure, which will be systematically studied in the future.

An interesting issue is the electronic topological transition in antimony chalcogenides like Sb_2_S_3_ and Sb_2_Se_3_. Those chalcogenides have the same layered structure at ambient conditions. Both of them were reported to undergo electronic topological transitions at a few GPa^30,32,38,48^, at which the atomic vibration and lattice compressibility exhibit sharp anomaly. The transition occurs when a band extreme associated with a Van Hove singularity passes through the Fermi level and causes a strong redistribution of the density of electronic states near the Fermi surface. The density of states as a function of pressure over the entire range of pressure is provided in Supplementary Fig. 5. Our work not only verified previous results on the compressibility and vibrational modes, but also, observed the onset of band gap energy discontinuity at ~4 GPa, along with the indirect-direct type of band gap. This is probably caused by the charge density redistribution from the electronic topological transition. The indirect-direct bandgap transition of Sb_2_S_3_ was previous reported in Ibañez et al.^33^ and was verified by our hybrid function simulation. This bandgap transition was achieved by the opposite shifts of the conduction band minima at the Z and Γ points in the Brillion zone. While the *d*-electrons are deep in the valence state of Sb_2_S_3_ and the electronegativity of S atoms are stable in the pressure range of 0-11 GPa (Supplementary Fig. 6), the abnormal shifts in the conduction band is mainly a pressure effect, which reduces the interatomic distances and strengthens the atomic interactions, leading to the redistribution of energy bands. The critical transition pressure was then anchored to the formula used to calculate the band gap in our UV-Vis experiment.

We also conducted Bader charge analysis to confirm the transition at 11 GPa^49^. A sudden change of Sb and S charges was observed at the critical transition pressure. For example, charges of Sb(1) increased from 2.0 to 2.5 while that of S(1) dropped from 7.5 to 7.0. The transition came along with the disappearance of Sb^3+^ lone-electron pair and the formation of SbS_7_ polyhedra in the bulk structure. Pressure has been previously used to engineer the band gap energies of insulators and semiconductors, which also alters their electronic structures. For example, insulators like CsAuI_3_ perovskite can even reach a rate of 0.2 eV/GPa in closing its band gap. Our UV-Vis experiment showed a normalized 0.07(1) eV/GPa decrease of band gap energy, and this trend generally reflected the shortening of bond-length with more overlapped charge density between atoms (Fig. 3).

## Conclusion

In summary, the band gap engineering in Sb_2_S_3_ is achieved through two-step transitions at 4 and 11 GPa. The first bandgap transition is mainly due to the redistribution of charge density near the CBM. The latter is controlled by an isostructural phase transition, which is the result of collapsing layers. The evolution of band gap energies in compressed Sb_2_S_3_ is covered by this hierarchical electronic phase transition mechanism. Although compression alone could not overcome self-trapping or yield strong photoluminescence, future opportunities may lie in combined doping and pressurization methods to eliminate the harmful self-trapping states and optimize band structures^14,50,51^.

## Methods

### Sb_2_S_3_ single crystals

We use natural antimony (III) sulfide (Sb_2_S_3_) single crystal collected from Xikuangshan mountain, Hunan province, China. The natural crystals were polished and the chemical composition was measured by electron probe micro-analysis coupled with a scanning electron microscope (SEM-EPMA) available at the Central South University (Supplementary Fig. 7 and Supplementary Table 1). By averaging 8 spots on the polished single-crystal facet, the natural single-crystal sample exceeds 99% purity and its deviated atomic ratio of δ (Sb: S) value is below 0.08%. Single-crystal sample was then crushed into small pieces with typical size of 50 × 20 × 10 μm^3^ and arbitrary orientation before loading into the diamond anvil cell (DAC).

### X-ray diffraction experiments

Single-crystal x-ray diffraction experiments were performed at 13BM-C station of GeoSoilEarthCARS (GSECARS) of the Advanced Phonon Source, Argonne National Laboratory. The crushed natural stibnite (Sb_2_S_3_) single-crystal sample was loaded in between two diamond anvils of 400 μm culet size and was sealed with a T301 steel gasket. The sample chamber was a drilled hole of 200 μm diameter with neon gas as the pressure medium. Pressure was applied by increasing pressure in a gas memberane system that coupled with the DAC. The monochromatic beam wavelength used for data collection was 0.434 Å with a focus spot of 12 × 18 μm^2^. Single-crystal diffraction patterns were collected on a Pilatus detector at each x-ray incident angle (1^o^ per image) from −30^o^ to 30^o^ for 10 s each. The orientation matrix, the diffraction data reduction, and crystal structure refinement were performed using the APEX3 program (Bruker). The lattice parameters from powder XRD patterns were intially reduced by the software Unitcell^52^. Then their atomic positions are refined by the program GSAS. Pressure is primarily determined by calibrating the ruby fluorescence line shift in an online Ruby system. The pressures are also compared to the equation of state of neon, from which measurement uncertainties <1 GPa are achieved throughout the experiment.

### Low-frequency Raman spectroscopy

In situ high-pressure Raman measurements were conducted on a customized system available at Center for High Pressure Science and Technology Advanced Research (HPSTAR). Spectrums are taken for the back-scattering geometry using an Argon laser (532 nm and power <1 mW) in the range 0-500 cm^−1^ with a spectral resolution of 1.0 cm^−1^, and the resolution of the laser spot is ~10 μm. The acquiring time for each spectrum was 60 s and each collection was repeated for 10 times to attenuate the effects of fluorescence and cosmic rays. Raman spectra were fitted by a Lorenz-type function using Peakfit v4.12 software to determine the positions of each Raman mode.

### In situ high-pressure photoluminescence measurement

In situ high-pressure photoluminescence measurements were conducted on a customized system available at the HPSTAR. To measure the high-pressure optical properties (e.g., UV-Vis absorption spectroscopy and photoluminescence) of Sb_2_S_3_ in an diamond anvil cells (DACs), we used low fluorescence type IIa diamonds with a culet size of 300 μm. Absorption spectra were collected using a Xeon light source between 320 and 1600 nm. The absorption spectra and optical images were measured in a home-designed spectroscopy system in a micro-region (Gora-UVN-FL, built by Ideaoptics, Shanghai, China). Silicone oil was used as the pressure transmitting medium.

### First-principles calculation

First-principles calculations were performed under the framework of density functional theory through Vienna’s Ab Initio Package ver. 5.3.4. The generalized gradient approximation of Perdew, Burke, and Ernzerhof revised for solids was implemented to describe the exchange-correlation functions^53,54^. Pseudopotentials were used with eight valence electrons for Sb atoms (4s^2^4p^3^) and six for S atoms (3s^2^3p^4^). We employed a 2 × 7 × 2 *k* point mesh. The structures were allowed to relax for cell variables, cell volume, and atomic positions. We applied a simple D2 method of Grimme^55^ with a 50 Å cutoff radius to calculate the long-distance van der Waals correction. A plane-wave basis set with a kinetic energy cutoff of 350 eV was found sufficient to converge the force between atoms less than 0.01 eV/Å. After structural relaxation, the density of stateas and band structures are re-calcualted using the HSE06 hybrid functional^56^.

For simulated Raman spectroscopy, we first calculated the vibrational modes of Sb_2_S_3_ unit cell using a density functional perturbation theory. The Raman off-resonant activity of each vibrational mode was then computed the derivative of the polarizability through the program Raman-sc, and the source code for the algorism is available at https://raw.githubusercontent.com/raman-sc/.

## Supplementary information

Supplementary Information

Description of Additional Supplementary Files

Supplementary Data 1

## Data Availability

The authors declare that all data supporting the findings of this study are available within the paper and its supplementary files (Supplementary Information and Supplementary Data 1). The data supporting the findings of this study have been deposited at the 4TU Center for Research Data (10.4121/15131655). Any additional data can be requested by e-mailing the corresponding author. Source data are provided with this paper.
