# Supplementary material for: Phase transition mechanism and bandgap engineering of Sb2S3 at gigapascal pressures
Source: Commun Chem. 2021 Sep 2;4:125. doi: 10.1038/s42004-021-00565-4 (PMC9814834; doi:10.1038/s42004-021-00565-4)
Supplement: Supplementary file 1 — Supplementary Information [file 42004_2021_565_MOESM1_ESM.docx]

Supplementary Information for

Phase transition mechanism and bandgap engineering of Sb_2_S_3_ at gigapascal pressures

Zhongxun Cui^1,3^, Kejun Bu^1^, Yukai Zhuang^1^, Mary-Ellen Donnelly^1^, Dongzhou Zhang^2^, Philip Dalladay-Simpson^1^, Ross T. Howie^1^, Jiandong Zhang^3^, Xujie Lü^1^, Qingyang Hu^1,4*^

^1^Center for High Pressure Science and Technology Advanced Research, Shanghai 201203, P.R. China

^2^Hawai’i Institute of Geophysics and Planetology, School of Ocean and Earth Science and Technology, University of Hawai’i at Manoa, Honolulu, HI, 96822, USA

^3^Key Laboratory of Metallogenic Prediction of Nonferrous Metals and Geological Environment Monitor, Ministry of Education, Central South University, Changsha 410083, P.R. China

^4^CAS Center for Excellence in Deep Earth Science, Guangzhou Institute of Geochemistry, Chinese Academy of Sciences, Guangzhou 510640, P.R. China

^*^Corresponding author: Qingyang Hu (qingyang.hu@hpstar.ac.cn)


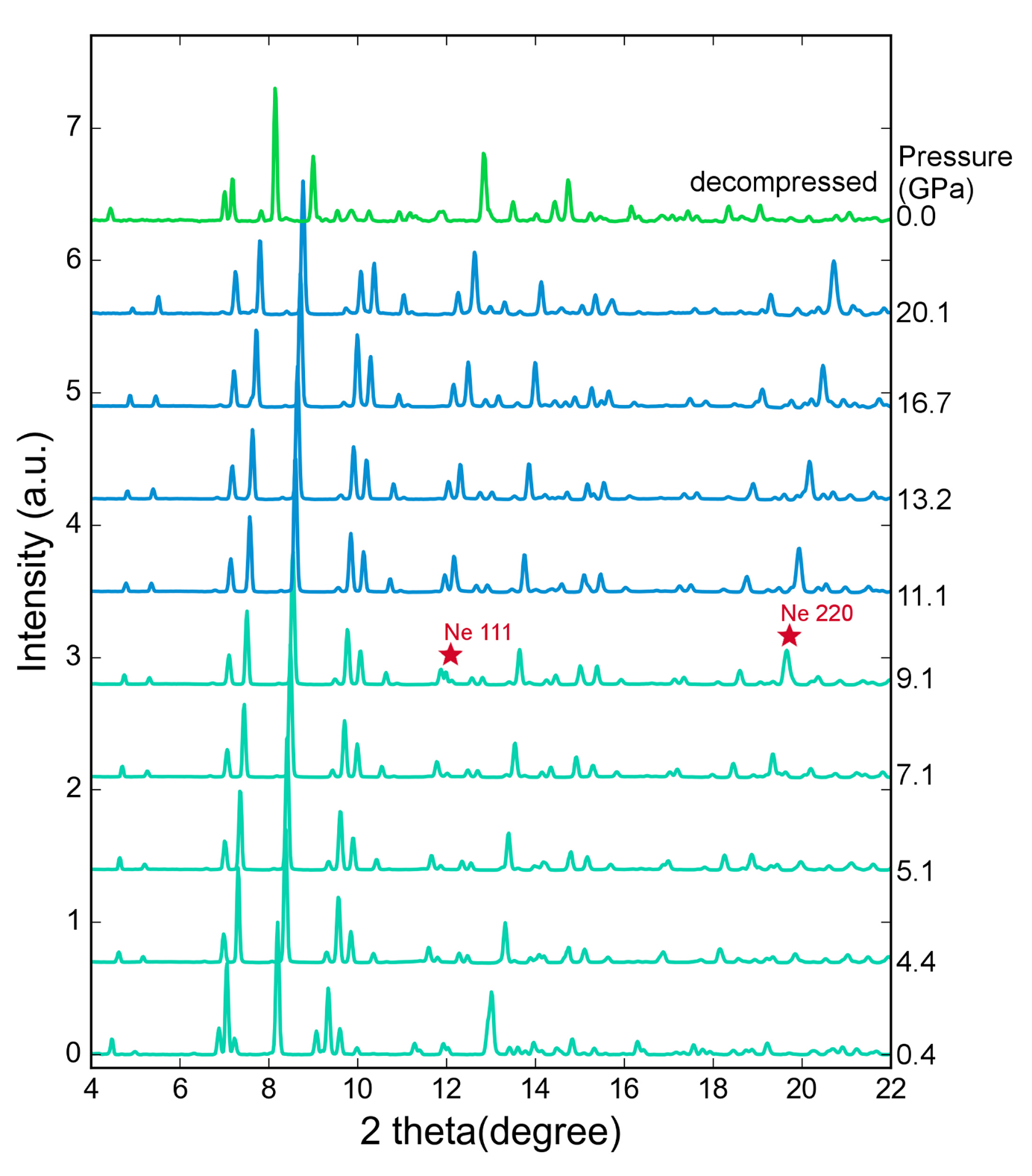


**Supplementary Figure 1. Integrated diffraction pattern of compressed Sb_2_S_3_.** The pressure media was neon and thus the sample chamber was under pseudo hydrostatic conditions. The neon peaks appeared and became prominent at around 9.1 GPa. Otherwise, the set of peaks were traceable to the *Pnma* space group throughout the studied range of pressure.


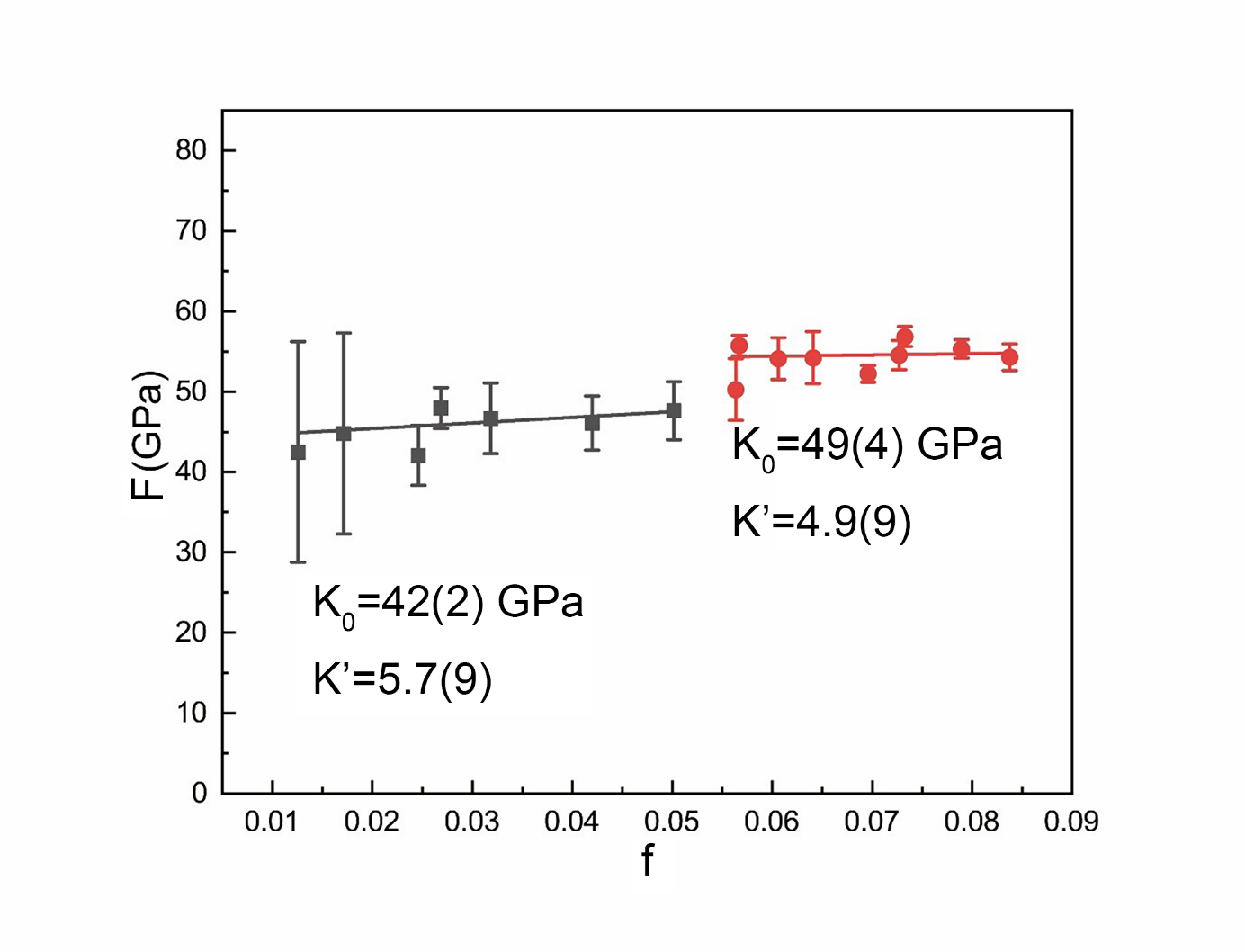


**Supplementary Figure 2. The F-f plot according to M. Hazen and R.T. Downs, Rev. Mineral. Geochem., 2000.** Here f is defined as $f=\left( {(V_{0}/V)}^{2/3}-1 \right)/2$ , $F=K_{0}+\left( 3/2 \right)K_{0}\left( K^{'}-4 \right)f$


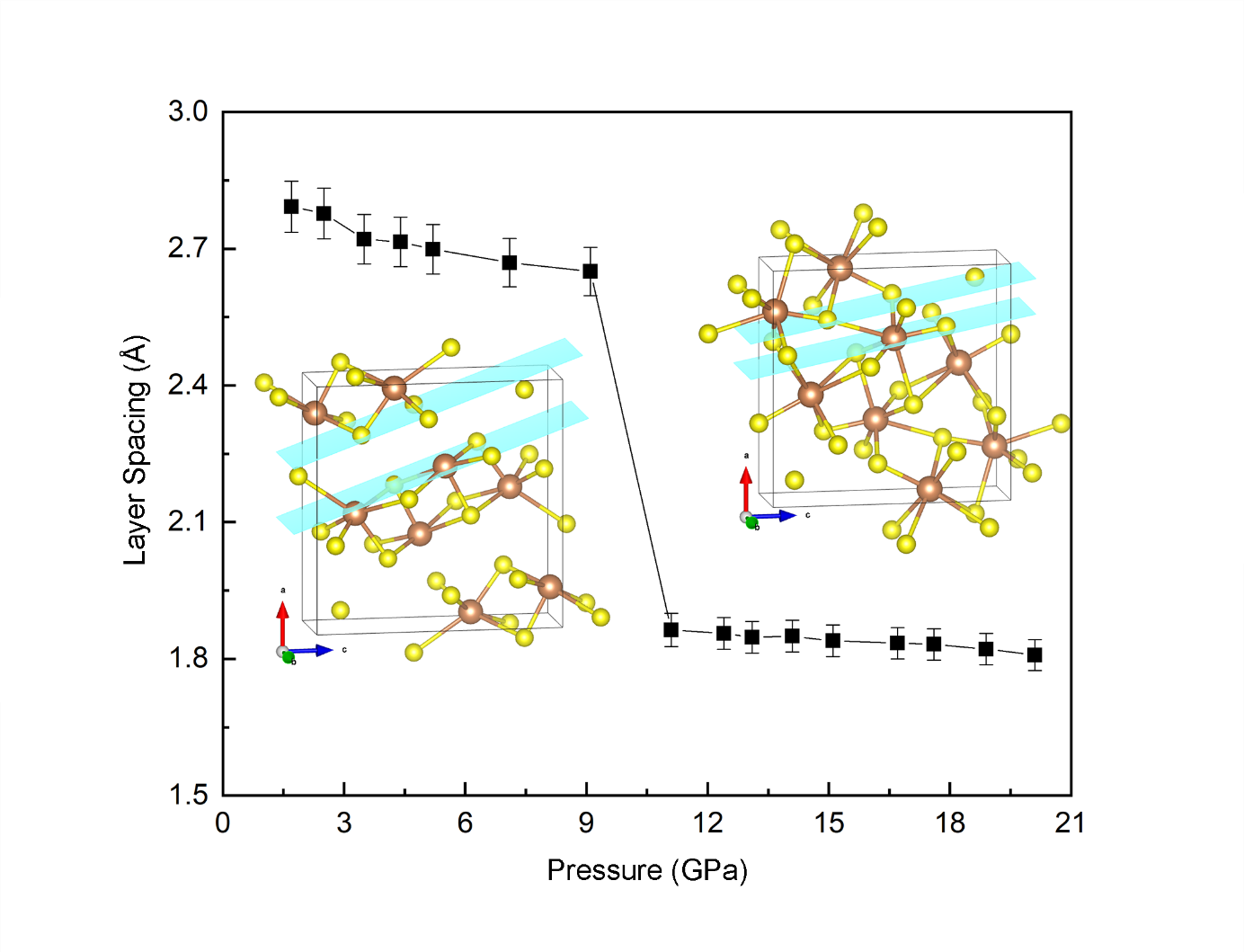


**Supplementary Figure 3. The layer spacing of compressed Sb_2_S_3_ layers**.


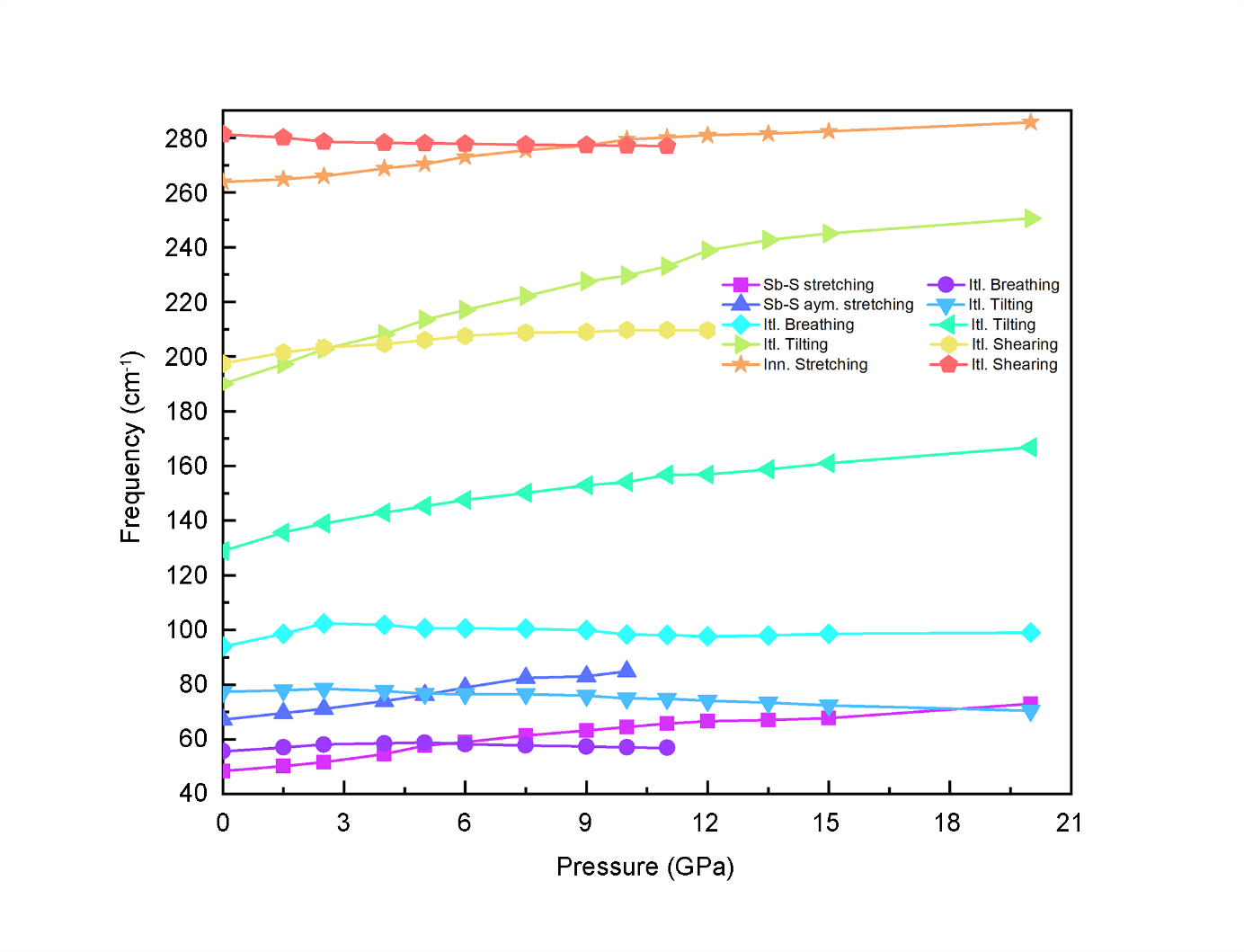


**Supplementary Figure 4.** **Mode displacements are derived from first-principles simulation at low pressure.** The vibrational motions of each mode were used to determine the vibration type.


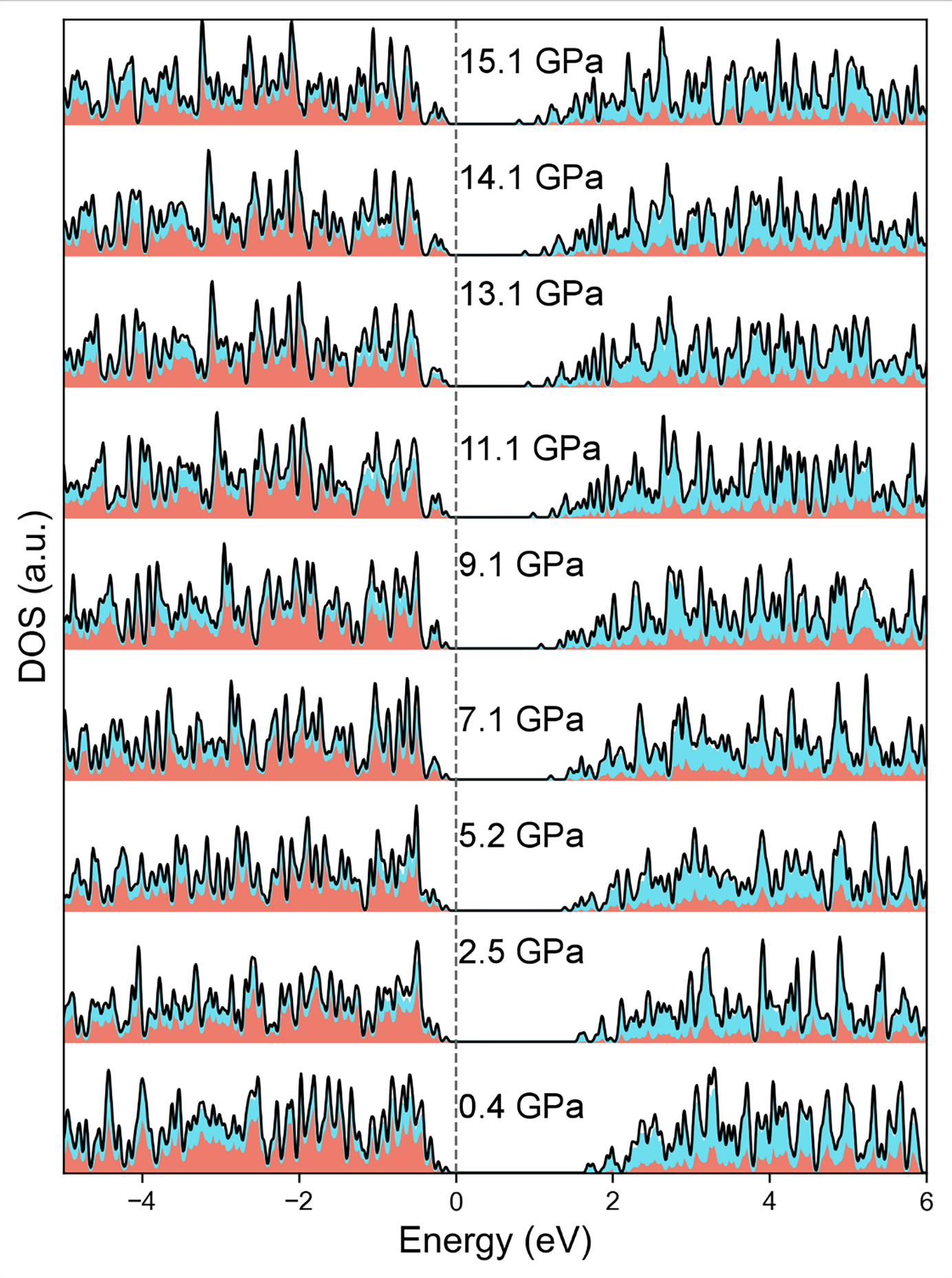


**Supplementary Figure 5. Mode displacements are derived from first-principles simulation at low pressure.** The lattice parameters were directly taken from our experiment. The fermi energies were aligned to 0 eV. Fill blue color and red color represent Sb 5*p*, and S 3*p* orbital projected DOS, respectively.


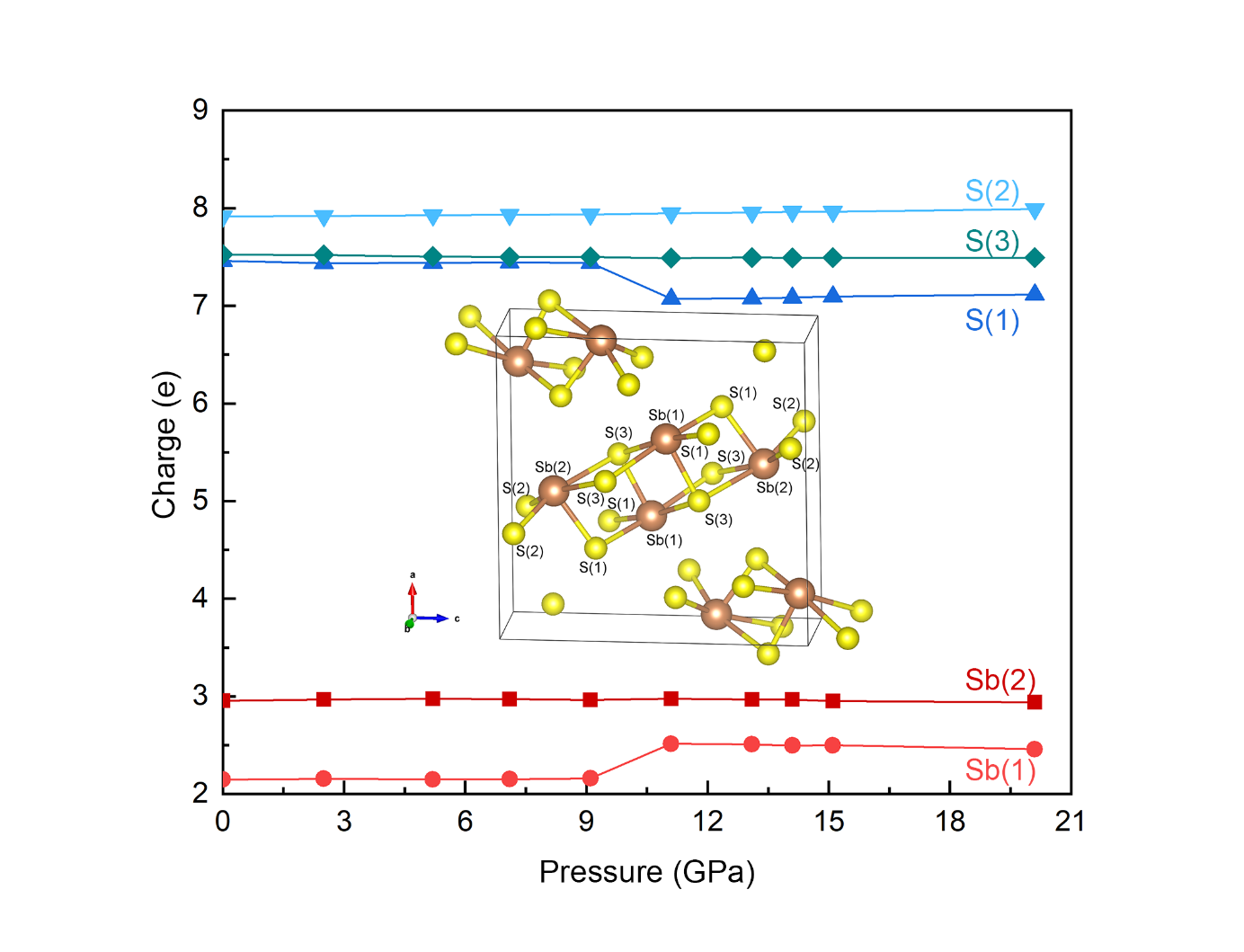


**Supplementary Figure 6. The evolution of Bader charges up to 20 GPa.** Since all atoms in Sb_2_S_3_ occupy 4c Wycoff position, we labeled the atoms in the numerical order. A sudden jump of Sb(1) charges and drops of S(1) charges were observed at the critical transition point.


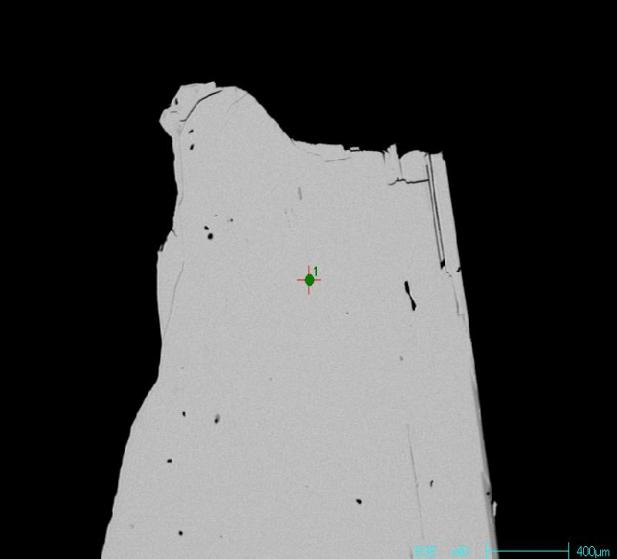


**Supplementary Figure 7. BSE image of polished Sb_2_S_3_ single crystals.**

**Supplementary Table 1. Chemical composition of Sb_2_S_3_ sample taken from electron probe micro-analysis.** The only tiny number of impure elements were found.

| # | **Sb La** | **S  Ka** | Fe Ka | As La | Cu Ka | Pb Ma | Zn Ka | Bi Ma | Hg Ma | Total | Sb:S | Δ (%) |
| --- | --- | --- | --- | --- | --- | --- | --- | --- | --- | --- | --- | --- |
| 1 | **71.6** | **28.1** | 0 | 0.97 | 0.03 | 0.02 | 0.06 | 0 | 0.05 | 100.8 | 0.670 | 0.32 |
| 2 | **71.3** | **28.2** | 0.02 | 1.08 | 0 | 0 | 0 | 0.02 | 0.03 | 100.7 | 0.665 | -0.17 |
| 3 | **71.3** | **28.0** | 0.02 | 0.89 | 0 | 0 | 0.09 | 0.14 | 0.12 | 100.5 | 0.669 | 0.28 |
| 4 | **71.2** | **27.8** | 0 | 0.91 | 0 | 0 | 0 | 0.03 | 0 | 99.9 | 0.674 | 0.72 |
| 5 | **71.4** | **27.9** | 0 | 1.02 | 0 | 0 | 0.06 | 0.08 | 0.05 | 100.5 | 0.673 | 0.66 |
| 6 | **71.0** | **27.9** | 0.01 | 0.87 | 0.07 | 0 | 0 | 0.17 | 0.02 | 100.1 | 0.669 | 0.24 |
| 7 | **71.3** | **28.0** | 0 | 0.85 | 0 | 0.02 | 0 | 0.15 | 0.02 | 100.3 | 0.672 | 0.49 |
| 8 | **71.2** | **28.1** | 0 | 0.96 | 0 | 0.06 | 0 | 0.13 | 0.07 | 100.5 | 0.666 | -0.04 |

**Supplementary Table 2. Single crystal refinement of Sb_2_S_3_ at 1.7 GPa**.

|  | Sb_2_S_3_ |
| --- | --- |
| *P, T* conditions | 300 K, 1.7 GPa |
| Crystal system | Orthorhombic |
| Space group | *Pnma* |
| *a* (Å) | 11.276(4) |
| *b* (Å) | 11.002(2) |
| *c* (Å) | 3.744(1) |
| *V* (Å^3^) | 464.48(22) |
| *Z* | 4 |
| *V*/*Z* | 120.3 |
| *F*(000) | 600 |
| Wavelength | 0.4344 Å |
| 2 theta range (°) | 1.403 - 32.479 |
| Index ranges | -3≤*h*≤5 |
|  | -4≤*k*≤4 |
|  | -12≤*l*≤12 |
| Reflections collected | Unique reflections found: 170, 158 |
|  | Unique reflections possible: 628, 506 |
| Independent  reflections/ *R*_int_ | 170 / 0.0228 |
| Refinement method | Full matrix least squares on *F*^2^ |
| Data / restraints / parameters | 170 / 0 / 16 |
| Goodness of fit on *F*^2^ | 1.594 |
| Final *R* indices [I > 4σ(I)] | 0.0364 / 0.1812 |
| *R*_1_ / *wR*_2_ |  |
| *R* indices (all data) | 0.0425 / 0.1812 |
| *R*_1_ / *wR*_2_ |  |
| Largest diff. peak /hole (*e* / Å^3^) | 0.96 / -1.04 |
