# Supplementary material for: Phase transition mechanism and bandgap engineering of Sb2S3 at gigapascal pressures
Source: Commun Chem. 2021 Sep 2;4:125. doi: 10.1038/s42004-021-00565-4 (PMC9814834; doi:10.1038/s42004-021-00565-4)
Supplement: Supplementary file 2 — Description of Additional Supplementary Files [file 42004_2021_565_MOESM2_ESM.pdf]

# Description of Additional Supplementary Files

**File Name:** Supplementary Data 1

**Description**: A supplementary data file containing all our experimental and computational data.
